# Polyunsaturated Fatty Acids and Their Metabolites in Hyperemesis Gravidarum

**DOI:** 10.3390/nu12113384

**Published:** 2020-11-04

**Authors:** Richard Lindberg, Maria Lindqvist, Miles Trupp, Marie-Therese Vinnars, Malin L. Nording

**Affiliations:** 1 Department of Chemistry, Umeå University, 901 87 Umeå, Sweden; richard.lindberg@umu.se; 2 Department of Nursing, Umeå University, 901 87 Umeå, Sweden; maria.lindqvist@umu.se; 3 Department of Clinical Sciences, Obstetrics and Gynecology, Umeå University, 901 87 Umeå, Sweden; marie-therese.vinnars@umu.se; 4 Department of Clinical Sciences, Neurosciences, Umeå University, 901 87 Umeå, Sweden; miles.trupp@umu.se; 5 Division of Obstetrics and Gynecology, Örnsköldsvik Hospital, 891 89 Örnsköldsvik, Sweden

**Keywords:** omega-3, bioactive lipid, fatty acid, lipid mediator, eicosanoid, oxylipin, prostaglandin, hyperemesis gravidarum, nausea and vomiting of pregnancy, NVP

## Abstract

Polyunsaturated fatty acids (PUFAs) have been studied in relation to pregnancy. However, there is limited knowledge on PUFAs and their metabolites in relation to hyperemesis gravidarum (HG), a pregnancy complication associated with nutritional deficiencies and excessive vomiting. In order to survey the field, a systematic review of the literature was performed, which also included nausea and vomiting of pregnancy (NVP) due to its close relationship with HG. In the very few published studies found, the main subjects of the research concerned free fatty acids (four records), lipid profiles (three records), and bioactive lipids (one article about prostaglandin E_2_ and one about endocannabinoids). The authors of these studies concluded that, although no cause-and-effect relationship can be established, HG is linked to increased sympathetic responsiveness, thermogenic activity and metabolic rate. In addition, NVP is linked to a metabolic perturbance (which lasts throughout pregnancy). The low number of retrieved records underlines the need for more research in the area of PUFAs and HG, especially with regard to the underlying mechanism for the detected effects, potentially involving growth differentiation factor 15 (GDF15) since evidence for GDF15 regulation of lipid metabolism and the role for GDF15 and its receptor in nausea and vomiting is emerging.

## 1. Introduction

**Figure 1:**
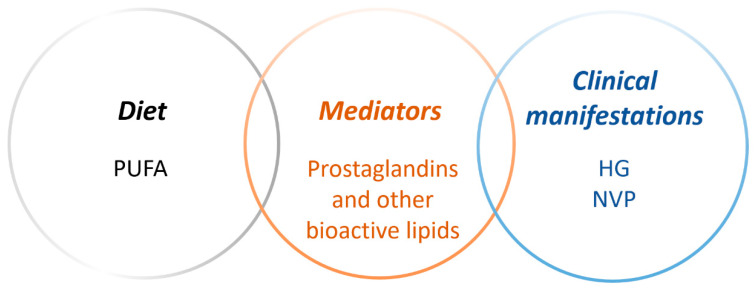
The aim of this review was to investigate links in the literature between dietary polyunsaturated fatty acids (PUFAs) and hyperemesis gravidarum (HG), as well as nausea and vomiting of pregnancy (NVP), with focus on bioactive lipid mediators in metabolic pathways of different PUFA precursors.

To maintain a healthy pregnancy, it is important to meet increased nutritional needs. Fetal development requires an adequate maternal supply of nutrients that influence both fetal growth and organ development throughout gestation [1]. In particular, the fetus depends largely on the maternal supply of polyunsaturated fatty acids (PUFAs) such as docosahexaenoic acid (DHA) and arachidonic acid (AA) via the placenta [2]. The fetal availability of PUFAs depends on both placental transport and metabolism [3]. Factors influencing the ability of the placenta to mediate the transfer of nutrients from the mother to the fetus include quality of placentation, area available for exchange, placental metabolism and placental blood flow [4]. Impaired placental function is involved in obstetrical complications, such as preeclampsia, intrauterine growth restriction and gestational diabetes mellitus (GDM) [5]. Although the pathogeneses differ, they are associated with compromised PUFA transport [6], placental inflammation and oxidative stress [7]. Furthermore, the complication itself may also result in impaired maternal synthesis and metabolism of PUFA [8]. Hence, suboptimal PUFA availability and dysregulated metabolism might both contribute to, and be a result of, pregnancy complications. The already mentioned preeclampsia, intrauterine growth restriction and GDM are among the most well-studied complications in this context [9]. However, the less well characterized pregnancy-associated disease, hyperemesis gravidarum (HG), may also involve impaired placental PUFA transport and/or metabolism, since the hallmark of HG is severe nausea and vomiting with increased risk of compromised nutritional status as a result [10]. It has also been suggested that a diet rich in fish leads to a lower risk of developing HG [11]. If this is due to certain fatty acids or their metabolites are yet to be determined (Figure 1), they need to be measured and correlated with clinical manifestations of HG. In order to summarize current information in the field and identify gaps of knowledge, the aim of this review was to evaluate the available evidence on lipid status in HG under the hypothesis that perturbed PUFA metabolism, reflected by altered levels of prostaglandins and other bioactive lipid mediators, is associated with HG.

## 2. Hyperemesis Gravidarum

HG is an extreme form of nausea and vomiting of pregnancy (NVP) contributing to malnutrition and dehydration, with increased risk for gestational anemia, preeclampsia and other adverse maternal and birth outcomes [12]. Insufficient maternal weight gain due to HG has for instance been identified as a risk factor for small for gestational age infants and preterm delivery [13]. Children exposed to HG in-utero are at increased risk of neurodevelopmental delay and autism spectrum disorder [14,15,16]. Long-term maternal consequences of HG include depression and post-traumatic stress syndrome [17,18]. In rare cases, HG has been the cause of maternal and fetal death [19]. It presents in 0.3–10.8% of pregnancies and is, after preterm labor, the second leading cause for hospitalization during pregnancy [20,21,22,23]. The uncertainty in incidence reflects the lack of a consensus definition for HG, as well as limited research in the area.

The pathogenesis of NVP and HG is multifactorial, involving factors of genetic, endocrine and gastrointestinal origin [24]. Novel insight into the genetic contribution to HG was recently provided by a genome-wide association study identifying variants in and/or around the locus of the placenta and appetite genes coding for growth differentiation factor 15 (GDF15) and insulin-like growth factor binding protein 7 (IGFBP7) as risk factors for HG [25]. In pregnancy, *GDF15* is expressed by the placenta and the circulating levels of GDF15 (also known as MIC-1) increase during the first trimester and remain high until delivery [26]. Studies of serum levels of GDF15 and IGFBP7 in HG suggest that the proteins are significantly increased in these patients compared to healthy pregnant controls with normal NVP and no NVP, respectively [27]. Furthermore, elevated GDF15 levels at 15 weeks of gestation have been associated with vomiting and the use of antiemetic medication [28]. An investigation of the anorexic and emetic effects of GDF15 showed that blockade of its receptor GFRAL may serve as an antiemetic treatment and that GFRAL agonists can induce nausea and vomiting [29]. GDF15 regulates lipid metabolism [30,31] and, during fasting, increased GDF15 levels promote fatty acid β-oxidation and ketogenesis in the liver [32] and influence lipid homeostasis with implications on atherosclerotic plaque formation [33]. Furthermore, GDF15 may induce an aversive response to nutritional stress since it was found to trigger conditioned taste aversion in mice [34]. The same study showed that GDF15 levels increase in response to high-fat feeding in mice but display only modest changes in response to moderate caloric surpluses or deficits in mice or humans. Another study found that *GDF15* expression is increased in livers of non-alcoholic steatohepatitis (NASH) animal models and in humans with NASH due to diet-induced hepatic endoplasmic reticulum stress [35].

## 3. Polyunsaturated Fatty Acids and Bioactive Lipid Mediators in Pregnancy

**Figure 2:**
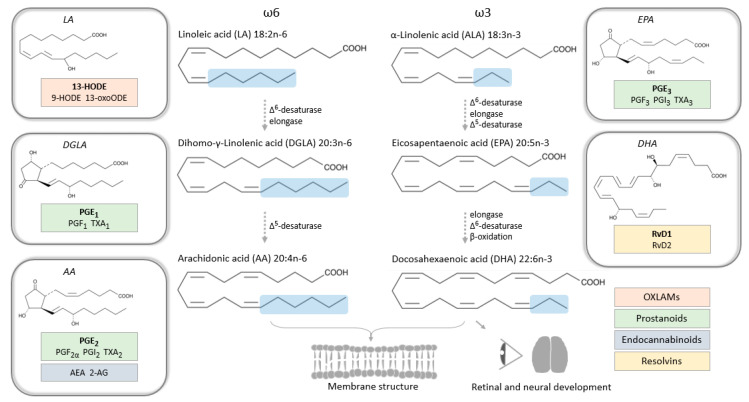
Biosynthesis of omega-6 and omega-3 PUFAs from dietary supplies in mammals, with examples of critical metabolites (bioactive lipids) in pregnancy framed in boxes. Abbreviations: HODE, hydroxyoctadecadienoic acid; oxo-ODE, oxooctadecadienoic acid; PG, prostaglandin; TX, thromboxane; AEA, arachidonoylethanolamide; AG, arachidonoylglycerol; Rv, resolvin; OXLAMs, oxidized linoleic acid metabolites.

Linoleic acid (LA, omega-6) and α-linolenic acid (ALA, omega-3) are dietary essential PUFAs from which AA (omega-6), DHA (omega-3) and eicosapentaenoic acid (EPA, omega-3) are derived (Figure 2). Human cells are unable to introduce double bonds in the fatty acid chain before carbons three (n-3) and six (n-6) from the n terminus, which means that AA (20:4n-6), DHA (22:6n-3) and EPA (20:5n-3) have to be formed by elongation and desaturation of the parent dietary compound, LA (18:2n-6) and ALA (18:3n-3), respectively. Fatty acids can circulate in free form, bound to albumin in the blood stream, or become incorporated into triglycerides and phospholipids, and then circulate with the aid of lipoprotein particles, also containing cholesterol esters, free cholesterol, and apolipoproteins. Lipoproteins are divided into several classes, such as high-density lipoproteins (HDLs) and low-density lipoproteins (LDLs), depending on size, lipid composition and type of apolipoprotein.

De novo lipid synthesis has been demonstrated in fetal liver tissue [36], but the fetus depends, to a large extent, on maternal dietary LA and ALA serving as precursors for omega-6 and omega-3 fatty acid synthesis in the maternal liver for sufficient fatty acid supply [37]. The transfer of PUFAs from mother to fetus is mediated by the placenta and there is a need for enzymes capable of lipolysis at the maternal–fetal interfaces presented by the placenta to support the transplacental transport of lipids contributed by maternal adipose tissue [38]. The placenta layer closest to maternal circulation is made up of trophoblasts called syncytiotrophoblasts, which constitute the transporting epithelium of the placenta that triglycerides cannot cross. Details on the transporting system have been reviewed elsewhere, for instance in Brett et al., who explore how pregnancy pathologies may affect transporter expression and activity [39].

The roles of PUFAs include providing energy and maintaining cell membrane structures, as well as functioning as precursors for bioactive lipid mediators. The latter is a collective name for eicosanoids (derived from AA), specialized pro-resolving mediators (SPMs), endocannabinoids, sphingolipids, isoprostanes and other fatty acid metabolites that serve as key pathophysiological mediators of multiple intra- and intercellular processes, including inflammation [40].

Eicosanoids include AA metabolites such as prostaglandins (PG) derived from the cyclooxygenase (COX) pathway, leukotrienes from the lipoxygenase (LOX) pathway and epoxyeicosatrienoic acids (EETs or EpETrEs) from the cytochrome P450 (CYP) pathway [41]. AA epoxides are further oxidized by soluble epoxide hydrolase to produce diols (DHETs or DiHETrEs) [42]. The same enzymes can oxidize other PUFA precursors and thereby produce metabolites collectively known as oxylipins, with the same functional groups as AA-derived metabolites, but with different fatty acid chain length [43]. In the developing brain (studied in rats), oxidized LA metabolites (so called OXLAMs) made up the majority of oxylipins in the unesterified pool, despite low relative abundance of their LA precursor; and the most abundant OXLAM (13-HODE) stimulated axonal outgrowth in primary cortical neurons [44]. In humans, it has been stipulated that LA intake is linked to abnormal neurodevelopment, but underlying mechanisms are unknown [45]. It has been shown that the ratio of maternal intake of omega-6 to omega-3 PUFAs is negatively associated with both mental and psychomotor development in infants at six months of age [46]. So, not only the absolute amounts of dietary PUFAs, but also the ratio between omega-6 and omega-3 PUFAs seems to be of importance for neurodevelopment; this is in accordance with other aspects of human health where beneficial effects of low omega-6 to omega-3 ratio have been observed [47].

Prostaglandins are perhaps the most noteworthy among the eicosanoids in pregnancy, since they are pro-inflammatory mediators known to evoke the onset of labor. Induction of labor at term in pregnancies by administration of prostaglandins (PGE_2_ and PGF_2_α) is common practice [48]. PGE_2_ and PGF_2_α have also been used for therapeutic abortion, dating back to the 1970s [49,50], as well as to treat postpartum hemorrhages [51]. Furthermore, there is an increased risk of malformations and miscarriage in early pregnancy associated with non-steroidal anti-inflammatory drugs (NSAIDs) targeting the COX enzyme and thereby inhibiting prostanoid (including prostaglandin) formation [52]. PGE_2_, PGF_2_α and other oxylipins have been studied in relation to preterm birth (reviewed by Aung et al. [53]), in association with gestational age in term pregnancies [54], as well as in relation to fetal growth [55].

SPMs, including resolvins, are a group of bioactive lipid mediators specifically derived from omega-3 fatty acids that are able to actively resolve inflammation [56,57]. Thereby, the protective effects of omega-3 fatty acids have, at least in part, been attributed to SPM activity, and it has been suggested that SPMs are important for a healthy pregnancy. In this context, resolvin D2 has been found to alter the inflammatory response in human placental tissue [58] and increased resolvin D1 and D2 levels have been associated with infant neonatal intensive care unit admission, while decreased levels were associated with term birth (≥36 weeks of gestation) [59].

Endocannabinoids are bioactive lipids that can bind to and activate the same receptors as the main psychoactive component of cannabis [40]. They are derived from PUFA precursors, and include arachidonoylethanolamide (AEA), also known as anandamide, derived from AA. In early pregnancy, AEA levels in blood are inversely correlated with levels of the AEA-degradation enzyme fatty acid amide hydrolase (FAAH) in peripheral blood mononuclear cells [60]. Furthermore, elevated AEA levels have been found in women who fail to achieve an ongoing pregnancy after in vitro fertilization treatment, and it has been suggested that low AEA levels are required for the pregnancy to progress successfully [60,61]. The link between AEA and fertility was corroborated by higher levels in the follicular phase vs. the luteal phase of the menstrual cycle, and trimester-dependent levels during pregnancy, with a dramatic increase during labor [62].

## 4. Results from Literature Search

**Figure 3:**
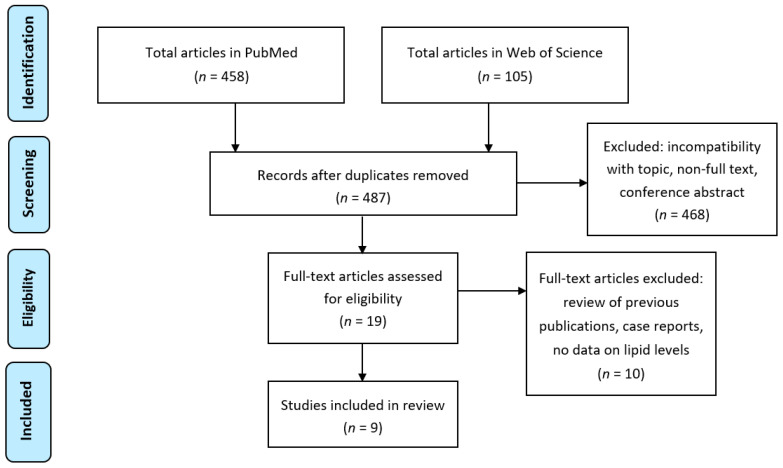
Flowchart of the retrieval of studies included in the review. The search terms were: “hyperemesis gravidarum”, “nausea and vomiting of pregnancy”, “fatty acid”, “PUFA”, “docosahexaenoic acid”, “eicosapentaenoic acid”, “arachidonic acid”, “eicosanoid”, “oxylipin”, “OXLAM”, “prostaglandin”, “bioactive lipid”, “lipid mediator”, “resolvin”, “endocannabinoid”, “lipid”, “phospholipid” and “sphingolipid”. Exclusion due to incompatibility with topic concerned studies related to medical treatments for incomplete miscarriage, termination of pregnancy, labor induction, acute fatty liver of pregnancy, liver disease in pregnancy, fertility control, marijuana use in pregnancy, contraceptive efficacy, dental practice, lipid peroxidation, antioxidant activity, thyroid function, total parenteral nutrition, mitochondrial beta-oxidation, oxidative stress and ginger. Full-text articles that were excluded concerned one review of previous publications (no new data), two case reports, one study on β-hydroxybutyrate, and the remaining six did not contain any data on lipid levels in samples from study participants. (PRISMA template for the flowchart was used [63].)

To establish the state of current published research on maternal PUFAs and their metabolite levels associated with HG symptoms, the Preferred Reporting Items for Systematic Reviews and Meta-Analyses (PRISMA) model [63] was followed in searching PubMed and Web of Science databases using combinations of the search terms “hyperemesis gravidarum”, and “nausea and vomiting of pregnancy” with “fatty acid”, “PUFA”, “docosahexaenoic acid”, “eicosapentaenoic acid”, “arachidonic acid”, “eicosanoid”, “oxylipin”, “OXLAM”, “prostaglandin”, “bioactive lipid”, “lipid mediator”, “resolvin”, “endocannabinoid”, “lipid”, “phospholipid”, “sphingolipid” (Figure 3). In total, 487 unique records were retrieved, of which nine were relevant to findings that were within the topic of this review (Table 1), and thereby used for making recommendations on future research to determine if maternal PUFA supplementation and metabolism are correlated with or ameliorate HG symptoms.

Serum lipid profiles and apolipoprotein patterns have been studied in relation to HG and NVP by Aksoy et al. [64], Üstün et al. [71] and Järnfelt-Samsioe et al. [69]. In these studies, lipid profiles including serum levels of triglycerides, phospholipids, HDL cholesterol, LDL cholesterol, total cholesterol and apolipoprotein (apo)-A and -B, were analyzed. In the earliest record, by Järnfelt-Samsioe et al., 98 healthy pregnant women were studied in early and late pregnancy, and a different lipid content of LDL and HDL was discovered in women with NVP, both in early (*n* = 60) and late pregnancy (*n* = 53) [69]. Furthermore, in early pregnancy serum levels of total cholesterol were higher in women with NVP vs. non-pregnant controls, VLDL phospholipids were elevated in women with NVP compared to both control groups (pregnant and non-pregnant women), LDL free and total cholesterol levels were lower in women with NVP vs. non-pregnant controls, LDL levels of free and total cholesterol as well as phospholipids were higher in women with NVP vs. pregnant controls, HDL lipid fractions were higher in women with NVP vs. non-pregnant controls (but phospholipids and free cholesterol levels were lower than in pregnant controls). In late pregnancy, serum levels of total cholesterol and triglycerides were higher in previous NVP patients vs. pregnant controls and HDL triglyceride as well as phospholipid levels were higher in previous NVP patients vs. pregnant controls. The authors concluded that there is a metabolic difference between women with and without NVP, supported by distinct differences in serum lipids and lipoproteins, which lasts throughout pregnancy [69]. In Üstün et al., women with HG (*n* = 35) displayed lower levels of serum total cholesterol, HDL cholesterol, LDL cholesterol, apo-A and apo-B compared to healthy pregnant women (*n* = 39) [71]. However, triglycerides were not significantly different between the groups. In contrast, Aksoy et al. found no difference in serum total cholesterol, LDL cholesterol and apo-B (also not in triglyceride) between HG cases (*n* = 36) and healthy pregnant controls (*n* = 36), while HDL cholesterol and apo-A1 were decreased in HG [64].

Free fatty acid levels of DHA, EPA, AA, and others, have been studied by Ulubay et al. [70], Asakura et al. 2000 [65], Asakura et al. 2003 [66] and Watanabe et al. [72]. The latter two were studies in relation to the thermogenic response, which has been suggested to reflect enhanced mobilization of free fatty acids from lipid stores [73]. Serum free fatty acids were measured in the first trimester of pregnancy (around gestational week nine) and compared between HG patients (*n* = 26) and healthy pregnant controls (*n* = 26) by Ulubay et al. [70]. Arachidic acid and behenic acid, saturated fatty acids with 20 and 22 carbons, respectively, were significantly different between the groups. Arachidic acid was decreased and behenic acid was increased in the HG group. Furthermore, in a study by Asakura et al., plasma levels of free fatty acids were elevated in HG patients (*n* = 80) around gestational week 12 compared to NVP patients (*n* = 30), and healthy pregnant women (*n* = 30), although NVP patients and healthy pregnant women were sampled at earlier time points in their pregnancy than HG patients [65]. To study the thermogenic response, serum free fatty acids were measured in HG patients (*n* = 24) and pregnant controls (*n* = 20) by Asakura et al. to investigate temperatures in the interscapular region containing “brown fat” [66]. Lipolysis induced by thyroid hormones in this region results in a thermogenic response, reflected in the temperature [74]. Serum free fatty acid levels were elevated in HG compared to pregnant controls and symptomatic improvement in the HG patients was correlated with decreased free fatty acid levels in 17 of the 24 patients after approximately two weeks of treatment with 2000–2500 mL of electrolyte solution by intravenous infusion and a multivitamin preparation containing thiamine (vitamin B1) until appetite was regained and eating and drinking were possible again [66]. The levels were around two-thirds the level at admission, and still higher than in controls. A closer study on the thermogenic response in patients with HG was performed by Watanabe et al. [72]. They measured free fatty acids in serum after cold stress (hand exposure to 15 °C water for 50 s) and compared the levels between pregnant women with and without HG (*n* = 13) [72]. An increase in free fatty acid levels was observed in study participants with fasting HG, and the thermogenic response was correlated with the extent of weight loss [72]. The authors conclude that although no cause-and-effect relationship can be established, the results link HG to increased sympathetic responsiveness, thermogenic activity and metabolic rate.

Evidence for research on bioactive lipid levels in relation to HG and NVP was found in two records, by Gadsby et al. [67] and Gebeh et al. [68]. Gadsby et al. studied PGE_2_ and its association with NVP (together with interleukin 1β (IL-1β), and tumor necrosis factor-α (TNFα), substances that are secreted by trophoblast cells of the chorionic villi of the placenta) based on the fact that PGE_2_ is a known emetic agent [67]. Two serum samples were collected from pregnant women (*n* = 18) around 8 weeks of gestation, one sample with nausea present (symptomatic) and one sample with no nausea (control) during a 24-h time period. The study design was supported by the episodic nature of NVP, with 5% of women experiencing only one episode of nausea per day, while the rest having symptoms both before and after midday, and with 56% of the study participants having days with three or more episodes of nausea at some point during pregnancy [75]. Of the investigated substances (PGE_2_, TNFα and IL-1β) only PGE_2_ was associated with the presence of NVP at the time of sample collection. PGE_2_ levels were increased in symptomatic serum samples, independent of the time of day that the sample was collected [67]. The authors conclude that PGE_2_ is a possible causative agent for NVP. Another set of bioactive lipids was studied by Gebeh et al. [68]. They measured the endocannabinoid AEA derived from AA, as well as its shorter analogs oleoylethanolamide (OEA), with an 18 monounsaturated omega-9 carbon chain, and palmitoylethanolamide (PEA), with a 16 saturated carbon chain, in HG patients (*n* = 15) and healthy pregnant controls (*n* = 30) around seven weeks of gestation [68]. Furthermore, the association with markers for dehydration (hematocrit, serum urea and sodium levels) was investigated. Neither AEA, nor OEA and PEA, were found to be significantly altered in HG pregnancies, and there was no correlation with dehydration markers.

## 5. Discussion

Diets high in omega-3 fatty acids are considered beneficial to human health, including in relation to pregnancy outcomes, which was studied in a Cochrane review by Middleton et al. [76]. They found that preterm birth (<37 weeks of gestation) and early preterm birth (<34 weeks of gestation) were reduced in women receiving omega-3 PUFA compared with no omega-3. Furthermore, the results indicated a reduced risk of neonatal care admission and of perinatal death, as well as a reduced risk of low birthweight babies, but a small increase in large-for-gestational age babies. However, the effects on the risk for developing HG is not well documented, beside the previous mentioned study by Haugen et al., where it was suggested that a diet that includes fish is associated with a lower HG risk [11]. Furthermore, the risk of hospitalization for NVP was found in another study to increase with a higher daily intake of total fat, especially saturated fat [77]. However, there is limited knowledge about how the effects are mediated. Bioactive lipids have been proposed as key mediators and studied in response to omega-3 supplementation in healthy volunteers [78], during pregnancy [79], in placenta [80] and in maternal and cord plasma [59,81], but never in omega-3 supplementation studies of HG.

It is possible that the beneficial effects of high PUFA intake and the undesirable effects of high saturated fat intake with regard to HG and NVP can be attributed to bioactive lipids, but we were not able to find any evidence for that in the literature. Hence, more research is needed to establish maternal levels of PUFAs and bioactive lipids with potential impact on emesis, such as prostaglandins. In fact, already in early studies of PGE_2_ and PGF_2α_ for therapeutic abortion, adverse side effects were revealed along with uterine activity [49,50]. With regard to these side effects, Karim and Filshie noted that “The most common were nausea and vomiting, which occurred in 10 of the 52 cases and were found to be worse in patients already suffering from hyperemesis gravidarum.” [50]. The reassessment of these findings seems to have been neglected until Gadsby et al. designed a study to investigate the association between PGE_2_ levels and NVP [67], an investigation that needs to be extended to a cohort of HG patients in order to determine the relationship between PGE_2_ levels and HG.

With regard to the underlying mechanism of PGE_2_-induced emesis, GDF15 has been identified in a functional screen as NSAID-activated gene-1 (NAG-1) [82], but in a cell line apparently devoid of COX expression. It was postulated that NAG-1/GDF15 expression is activated by NSAID via a COX-independent pathway [83]. Contradictory to this hypothesis, GDF15 was shown to exhibit a reciprocal relationship with COX-2, with PGE_2_ suppressing GDF15 expression [84]. Together this suggests that further study of the interaction of GDF15 and COX pathways is warranted in the context of emesis, considering that elevated GDF15 levels have been associated with vomiting [28], and that variants of *GDF15* have been identified as risk factors for HG [25]. The hypothesis that PGE_2_ causes NVP is contradictory to the finding that NVP is associated with lower rates of pregnancy loss [85] and that PGE_2_ is used to induce pregnancy termination.

In Watanabe et al. [72], an increase in free fatty acid levels was observed in study participants with fasting HG, and the thermogenic response was correlated with the extent of weight loss. Although no cause-and-effect relationship was established, the results linked HG to increased sympathetic responsiveness, thermogenic activity and metabolic rate. In mice, oxygen consumption and heat production were lower in GDF15 (−/−) male mice, indicating a lower metabolic rate; whereas a lower metabolic rate also was reported in GDF15 (−/−) female mice, the difference was not statistically significant [86]. Taken together, this corroborates the role of GDF15 in lipid metabolism and the fact that HG has been linked to alterations in metabolic rate.

In six of the retrieved records, enzymatic or immunoassay measurement principles were utilized, which is a relatively simple and at the same time sensitive method for investigations of lipid and lipoprotein metabolism to indicate liver dysfunction [69], endothelial cell dysfunction [71], oxidative stress [64] and lipolytic activity [72]. The measurement techniques were appropriate for the research questions under study in all of these studies. However, in future studies of bioactive lipids mediating the effects, alternative methodologies will be required to reach sufficient quality for these types of analytes in order to map the PUFA metabolic pathways with adequate resolution. Furthermore, lipoprotein fractionation and compositional analysis of fatty acids within different lipid classes are sensitive towards sample handling and storage [87], further emphasizing the need for stringent analytical protocols in studies of lipid profiles. Metabolomics and, in particular, lipidomics, may offer the platform needed for such analyses since it gives information closely related to disease phenotype [88]. Mass spectrometry-based metabolomics methods have been used in several studies of the early-life environment, for instance in Martens et al. [89] and Gouveia-Figueira et al. [54]. These studies demonstrate practical examples of successful mass spectrometry protocols that can be used for the benefit of PUFA and HG research; for instance, in testing our hypothesis that perturbed PUFA metabolism, reflected by altered levels of prostaglandins and other bioactive lipid mediators, is associated with HG.

A suitable study design to investigate the association between PUFAs and the reduction rate of HG could, for instance, consist of a randomized controlled trial (RCT) allocating PUFA supplementation to one group of HG patients, and placebo to another group of HG patients. Further studies on the impact of this intervention on PUFA metabolism in relation to HG would require measurements of plasma levels of precursor fatty acids and their metabolites in association with clinical manifestations of HG among the study participants. PUFA intervention warrants further study, however, an RCT involves large amounts of resources. So, enrollment of HG patients into a smaller pilot study prior to considering a large-scale comparative multiple center study would be the most viable option in terms of an RCT interventional pharmacological study of PUFAs.

In addition, a case-control study of recalled dietary records prior to onset of HG could contribute with important information on a potential association between an imbalance of PUFAs and onset of HG, accompanied by measurements of precursor fatty acids and their metabolites in the blood stream for interrogations of dietary effects on circulating levels. The history of exposure to PUFAs among the cases with HG compared to a comparison group consisting of individuals without HG (controls) could provide an estimate of PUFA levels associated with HG. Measured levels of PUFA metabolites could add insight into the underlying mechanism mediating the effect.

Furthermore, the relative risk of developing HG could be investigated in a cohort study following individuals over time while recording their PUFA intake and health status. However, the expected low incidence rate for HG would require a very large cohort in order to enable comparisons of the occurrence of HG based on dietary habits among the study participants. Additional value could be added to such a cohort study by measurements of fatty acid precursors and their metabolites in the blood stream of a subset of study participants in correlation with their symptoms of HG, in line with the study design of Gadsby et al. [67]. They established an association between PGE_2_ levels and episodes of nausea in NVP, which supports the relevance of bioactive lipids, and thereby PUFA metabolism, in HG.

## 6. Concluding Remarks

Nutritional deficiencies and excessive vomiting are common in HG. As the placenta develops during pregnancy, GDF15 levels increase. Evidence for GDF15 regulation of lipid metabolism and the role for the GDF15/GFRAL axis in nausea and appetite regulation is emerging. Pharmacological modulation of GFRAL signaling is the target of several drug development programs including for obesity, cachexia and other eating disorders. Research on PUFAs and their metabolites in the context of HG suggests that there is a link between lipid metabolism and clinical manifestations of HG and NVP. However, the low number of retrieved records in this systematic review underline the need for more research in the area of PUFAs and HG, especially in relation to the importance of bioactive lipid mediators and *GDF15* expression. Robust analytical methods to accurately quantify lipid profiles will be an important driver for the development of this research field.

## Figures and Tables

**Table 1:** Retrieved records from PubMed and Web of Science databases.

Author, Year, Country	Study Participants		Analyte, Type of Lipid, PUFA or PUFA Metabolite	Analytical Method	Outcome
	Cases	Controls			
Aksoy et al., 2009, Turkey [64]	HG ^1^ patients (*n* = 36)	Healthy pregnant women (*n* = 36)	Serum TG, cholesterol, HDL, LDL, apo- A1 and -B	Enzymatic	TG, LDL-cholesterol, and apo B levels were not different; HDL-cholesterol and apo A1 were lower in HG vs. healthy pregnant controls.
Asakura et al., 2000, Japan [65]	HG patients (*n* = 80) and NVP (*n* = 30)	Healthy pregnant women (*n* = 30)	Plasma or serum free fatty acids	No info	Elevated free fatty acids in HG patients vs. healthy pregnant controls.
Asakura et al., 2003, Japan [66]	HG patients (*n* = 24)	Healthy pregnant women (*n* = 30)	Serum free fatty acids	Enzymatic	Elevated free fatty acids in HG patients vs. healthy pregnant controls and decreased free fatty acid levels in correlation with symptomatic improvement.
Gadsby et al., 2000, U.K. [67]	NVP symptomatic (*n* = 18)	NVP symptom-free (*n* = 18)	Serum PGE_2_	Radio-immunoassay	Increased maternal serum levels of PGE_2_ during episodes of nausea.
Gebeh et al., 2014, U.K. [68]	HG patients (*n* = 15)	Healthy pregnant women (*n* = 30)	Plasma AEA, OEA, PEA	LC-MS/MS	No differences in levels between the groups and no correlation with dehydration markers.
Järnfelt-Samsioe et al., 1987, Sweden [69]	NVP in early (*n* = 60) and late (*n* = 53) pregnancy	Healthy pregnant women in early (*n* = 38) and late (*n* = 34) pregnancy, non-pregnant controls (*n* = 22)	Serum TG, cholesterol, phospholipids, HDL, LDL, VLDL	Enzymatic	Early pregnancy: serum levels of TC, VLDL, and LDL were higher in NVP vs. non-pregnant controls; late pregnancy: higher serum levels of TC and TG in previously NVP patients vs. pregnant controls; HDL lipid composition was differential in both early and late pregnancy vs. controls.
Ulubay et al., 2017, Turkey [70]	HG patients (*n* = 26)	Healthy pregnant women (*n* = 26)	Plasma free fatty acids	GC	No difference in levels of DHA, EPA and AA. Higher behenic acid and lower arachidic acid in the HG vs. control group.
Üstün et al., 2004, Turkey [71]	Women with HG (*n* = 35)	Healthy pregnant women (*n* = 39)	Serum TG, cholesterol, HDL, LDL, apo-A and -B	Enzymatic	Lower levels of HDL cholesterol, LDL cholesterol and total cholesterol, apo-A and apo-B in the HG vs. control group.
Watanabe et al., 2003, Japan [72]	HG patients (*n* = 13) before and after challenge, fasting and non-fasting	Pregnant women with other obstetrical disease (*n* = 13)	Serum free fatty acids	Enzymatic	Free fatty acids increased in response to cold stimulus in fasting HG subjects, but not in the other groups.

^1^ Abbreviations: HG, hyperemesis gravidarum; PUFA, polyunsaturated fatty acid, NVP, nausea and vomiting of pregnancy; TG, triglyceride; HDL, high-density lipoprotein; LDL, low-density lipoprotein; VLDL, very low-density lipoprotein; TC, total cholesterol; apo, apolipoprotein; LC, liquid chromatography; MS, mass spectrometry; GC, gas chromatography; PG, prostaglandin; AEA, arachidonoylethanolamide; PEA, palmitoylethanolamide; OEA, oleoylethanolamide; DHA, docosahexaenoic acid; EPA, eicosapentaenoic acid; AA, arachidonic acid.
